# Effects of Iron-Oxide Nanoparticle Surface Chemistry on Uptake Kinetics and Cytotoxicity in CHO-K1 Cells

**DOI:** 10.3390/ijms17010054

**Published:** 2015-12-31

**Authors:** Camille C. Hanot, Young Suk Choi, Tareq B. Anani, Dharsan Soundarrajan, Allan E. David

**Affiliations:** Department of Chemical Engineering, Auburn University, Auburn, AL 36849, USA; thamarrys@hotmail.com (C.C.H.); yzc0036@auburn.edu (Y.S.C.); tba0008@tigermail.auburn.edu (T.B.A.); dks0009@tigermail.auburn.edu (D.S.)

**Keywords:** superparamagnetic iron-oxide nanoparticles (SPIONs), PEGylated nanoparticles, aminated nanoparticles, reproductive toxicity, nanotoxicity, uptake kinetics, ROS generation

## Abstract

Superparamagnetic iron-oxide nanoparticles (SPIONs) show great promise for multiple applications in biomedicine. While a number of studies have examined their safety profile, the toxicity of these particles on reproductive organs remains uncertain. The goal of this study was to evaluate the cytotoxicity of starch-coated, aminated, and PEGylated SPIONs on a cell line derived from Chinese Hamster ovaries (CHO-K1 cells). We evaluated the effect of particle diameter (50 and 100 nm) and polyethylene glycol (PEG) chain length (2k, 5k and 20k Da) on the cytotoxicity of SPIONs by investigating cell viability using the tetrazolium dye 3-(4,5-dimethylthiazol-2-yl)-2,5-diphenyltetrazolium bromide (MTT) and sulforhodamine B (SRB) assays. The kinetics and extent of SPION uptake by CHO-K1 cells was also studied, as well as the resulting generation of intracellular reactive oxygen species (ROS). Cell toxicity profiles of SPIONs correlated strongly with their cellular uptake kinetics, which was strongly dependent on surface properties of the particles. PEGylation caused a decrease in both uptake and cytotoxicity compared to aminated SPIONs. Interestingly, 2k Da PEG-modifed SPIONs displayed the lowest cellular uptake and cytotoxicity among all studied particles. These results emphasize the importance of surface coatings when engineering nanoparticles for biomedical applications.

## 1. Introduction

Iron-oxide nanoparticles, and in particular superparamagnetic iron-oxide nanoparticles (SPIONs), have gained a fair amount of attention in recent years, accounting for more than 5900 publications over the last five years (PubMed database, quick search in December 2015 with the following keywords: “iron-oxide nanoparticle”). Their magnetic properties, combined with the possibilities afforded by optimization of their surface chemistry, promise potential applications in many fields, including nanomedicine [1,2,3,4]. Iron-oxide nanoparticle formulations have already been approved by the U.S. Food and Drug Administration and the European Commission as contrast agents for magnetic resonance imaging (MRI) [5]. Additional applications being pursued include MRI-based cell tracking of SPION-loaded cells, magnetic field-directed stem cells for regenerative therapies, therapeutic magnetofection-based delivery of drugs or genes, and anti-tumor treatment with magnetic hyperthermia [2,5]. However, safety concerns and manufacturing challenges remain as significant hurdles to their development and clinical translation [5,6].

Although the overall safety of iron-oxide nanoparticles is generally accepted [7], the core and surface properties have the potential to trigger cytotoxicity once in contact with cells. Indeed, side-effects were reported in up to 23% of patients receiving Ferumoxtran-10, a dextran-coated SPION, as MRI contrast agent [8]. Although about 86% of cases were mild-to-moderate, some adverse reactions, such as anaphylactic shock, were life-threatening [9]. While the awareness of nanoparticle-induced toxicity is growing, toxicity studies represent only 11% of aforementioned publications on SPIONs over the last five years (PubMed, keywords’ iron-oxide nanoparticle toxicity’ in December 2015), and most of these studies were conducted with cancer cell lines. A thorough understanding of the physicochemical parameters underlying toxicity of SPIONs on normal systems is also essential, especially since repetitive administrations may be part of a diagnostic and/or treatment regimen.

Systematic studies are rare and the impact of SPIONs on the reproductive system in particular is poorly described. The number of results for the same quick search on PubMed with the following keywords: “iron-oxide nanoparticle toxicity reproductive” drops drastically to just 10 results. Moreover, toxicology data about the reproductive toxicity of SPIONs were mainly generated with *in vivo* studies using only dextran-coated SPIONs. Nevertheless, no effect on the fertility and reproductive performances of rats were observed with doses up to 17.9 mg Fe/kg/day of Ferumoxtran-10 (seven times the intended human clinical dose per administration) [10], but fetal skeletal and soft-tissue abnormalities were observed in rats and rabbits, and maternotoxicity in rabbits at doses above 15 mg Fe/kg/day [10]. In addition, bare ferric oxide nanoparticles, at concentrations above 10 mg Fe/L, have been shown to induce toxic effects on the early development of zebra fishes, causing hatchling delays, malformations, and mortality [11]. Mechanisms underlying these reproductive toxicities are poorly described, as is the actual distribution of SPIONs to reproductive organs.

Surface modification of SPIONs can influence the interactions between nanoparticles and cells [12,13,14,15]. Using bare SPIONs and SPIONs coated with –COOH or –NH_2_, it was demonstrated that nanoparticle surface properties induce different responses between various cell types, such as cell lines derived from the heart, brain, and kidneys [16]. Introduction of poly(ethylene glycol) (PEG) moieties onto the surface of SPIONs through covalent binding is commonly used to improve biocompatibility and reduce immunogenicity [5]. PEG molecules provide stability between the particles via steric repulsion [17] and seem to decrease adsorption of plasma proteins [18]. *In vivo*, PEGylated SPIONs generally have a longer plasma circulation half-life [19,20]. Nevertheless, conflicting results exist about the cytotoxicity of PEGylated particles. In a recent study, 2k Da-PEG SPIONs were shown to be more toxic than bare dextran SPIONs [19]; they were also taken up by cells to a greater extent compared to dextran-coated SPIONs. In another study, the viability of bovine vascular smooth muscle cells was improved when incubated for 24 h with 2k Da-PEG SPIONs, when compared with bare citric acid-coated SPIONs and SPIONs coated with 10k Da-PEG [21]. The toxicity of particles in these studies appeared to correlate well with their extent of cellular uptake [19,21] and the uptake process was highly dependent upon physicochemical properties of the core and coating [22,23].

The objective of this study was to assess the *in vitro* toxicity of SPIONs, with varying surface properties, on a cell line derived from a reproductive organ: Chinese Hamster Ovary (CHO-K1) cells. SPIONs evaluated in this study included particles with mean hydrodynamic diameters of approximately 50 and 100 nm (nominal size), with surface coatings that included starch, aminated-starch, and PEG. To further evaluate the impact of PEG molecules, three molecular weights of PEG (*i.e.*, 2k, 5k, and 20k Daltons; hereafter simply specified as 2k-PEG, 5k-PEG, and 20k-PEG, respectively) were used is this study. Cytotoxicity was determined *in vitro* with the tetrazolium dye 3-(4,5-dimethylthiazol-2-yl)-2,5-diphenyltetrazolium bromide (MTT) and the sulforhodamine B (SRB) assays. The cytotoxicity was then correlated with the overall cellular uptake kinetics and the generation of ROS.

## 2. Results and Discussion

### 2.1. Physicochemical Properties of Superparamagnetic Iron-Oxide Nanoparticles (SPIONs) Are Modified through Surface Functionalization

**Scheme 1:**
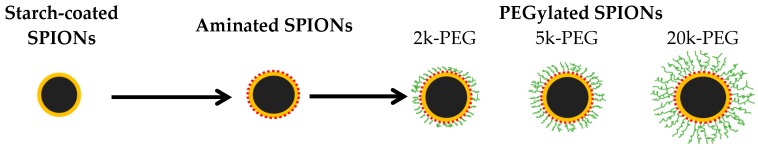
Surface modification of starch-coated superparamagnetic iron-oxide nanoparticles (SPIONs) into aminated and PEGylated SPIONs.

In the present study, the starch coating of 50 and 100 nm SPIONs was crosslinked and coated with amine groups, and then functionalized with NHS-polyethylene glycol (PEG) of varying molecular weight (*i.e.*, 2k, 5k, or 20k Da) as shown in Scheme 1.

PEG coatings are often utilized on nanoparticles for their good biocompatibility and favorable chemical properties that enable further modifications [24]. Since the coating itself may completely change the toxicity profile of SPIONs, SPIONs with a variety of surface coatings were tested, including starch-coated SPIONs, aminated SPIONs, and finally PEGylated SPIONs. Analysis of SPIONs by transmission electron microscopy (TEM) and Fourier transform infrared spectroscopy (FTIR), and measurement of their magnetic properties provided results similar to that already in the literature [20], confirming successful modification, and are not repeated here. Some physicochemical characteristics of the 10 different SPIONs tested, however, are summarized in Table 1 and Table 2.

**Table 1:** Mean hydrodynamic diameter (HD) and ζ-potential (ZP) of superparamagnetic iron-oxide nanoparticles (SPIONs) in deionized water and in Ham’s F-12K culture media supplemented with 10% FBS.

Sample	In Deionized Water, at 37 °C			In Supplemented Ham‘s F-12K Cultre Media, at 37 °C					
	HD (nm)	PdI	ZP (mV)	1 h		24 h		72 h	
				HD (nm)	PdI	HD (nm)	PdI	HD (nm)	PdI
50 nm SPIONs									
Starch-coated	47 ± 1	0.12	−23 ± 3	36 ± 1	0.25	68 ± 2	0.28	148 ± 4	0.30
Aminated	90 ± 2	0.18	+13 ± 1	57 ± 1	0.32	55 ± 5	0.38	50 ± 3	0.38
2k-PEG	72 ± 3	0.20	+36 ± 1	41 ± 1	0.30	40 ± 1	0.30	40 ± 1	0.31
5k-PEG	71 ± 1	0.20	+34 ± 1	37 ± 2	0.42	36 ± 2	0.41	36 ± 2	0.44
20k-PEG	72 ± 4	0.20	+33 ± 2	35 ± 1	0.48	36 ± 1	0.47	33 ± 2	0.47
100 nm SPIONs									
Starch-coated	92 ± 1	0.09	−4 ± 1	117 ± 1	0.29	461 ± 4	0.70	1133 ± 30	0.19
Aminated	127 ± 5	0.17	+43 ± 1	119 ± 4	0.30	110 ± 3	0.22	108 ± 3	0.22
2k-PEG	117 ± 2	0.14	+36 ± 1	93 ± 1	0.24	95 ± 3	0.23	95 ± 2	0.24
5k-PEG	126 ± 4	0.14	+37 ± 1	103 ± 2	0.25	102 ± 2	0.24	114 ± 2	0.29
20k-PEG	154 ± 5	0.12	+32 ± 1	122 ± 3	0.26	117 ± 3	0.26	146 ± 2	0.36

HD: hydrodynamic diameter (nm); ZP: ζ potential (mV); PdI: Polydispersity index; FBS: Fetal Bovine Serum; Results shown as mean ± std. error of mean.

The hydrodynamic diameters of both starch SPIONs were in close agreement with specifications provided by the manufacturer. After crosslinking of the starch coating and its amination, the mean hydrodynamic diameter of both 50 and 100 nm SPIONs increased significantly, possibly due to aggregation and/or due to loss of smaller particles during the processing. PEGylation further increased the hydrodynamic diameter as expected, but the effect of PEG molecular weight (2k, 5k, or 20k Da) varied between the 50 and 100 nm SPIONs. The effect on 50 nm particles was especially non-uniform as the aminated particles yielded the largest size, possibly due to aggregation. The hydrodynamic diameters of SPIONs were also evaluated when suspended in supplemented Ham’s F-12K culture media at 37 °C, the conditions employed for *in vitro* studies, with incubation times of 1, 24, and 72 h. The hydrodynamic diameters of starch-coated 50 and 100 nm SPIONs were observed to increase with time, whereas the size of aminated, 2k-PEG and 5k-PEG SPIONs, and 50 nm 20k-PEG SPIONs remained relatively constant over time. A moderate increase of the hydrodynamic diameter was noticed for 100 nm 20k-PEG SPIONs.

The surface charge of SPIONs gives an indication of their colloidal stability and may also further affect their cellular uptake. In one study, it was demonstrated that anionic nanoparticles, showing a high affinity for cell membranes, were captured more efficiently by cells than bare (dextran-coated) iron-oxide nanoparticles [25]. In deionized (DI) water, starch-coated SPIONs displayed a negative to near neutral ζ potential while the aminated and PEGylated particles showed a high positive surface charge. Surprisingly, a higher ζ potential was observed for the PEGylated 50 nm SPIONs compared to the aminated 50 nm SPIONs. The PEG layer would be expected to mask some of the surface charge and it is unclear why this was not observed, although measurements were repeated. Aggregation seen with the aminated SPIONs may have had some contribution to this result. 

Interestingly, the aminated SPIONs and PEGylated SPIONs had a similar amine content that was significantly greater than that of starch SPIONs, as shown in Table 2. Conversely, some variation was observed in the PEG content, with a general trend of decreasing PEG concentration as the PEG molecular weight was increased, probably due to increasing steric hindrance with the larger molecules. This tendancy, however, was less obvious with 100 nm SPIONs and the 2k and 5k Da PEGs.

**Table 2:** Amine- and PEG-content of SPIONs.

50 nm SPIONs			100 nm SPIONs		
Sample	Amine Content (mmol NH_2_/mg Fe)	PEG Content (nmol PEG/mg Fe)	Sample	Amine Content (mmol NH_2_/mg Fe)	PEG Content (nmol PEG/mg Fe)
Starch	0.14 ± 0.01	-	Starch	0.24 ± 0.01	-
Aminated	4.93 ± 0.01	-	Aminated	1.74 ± 0.01	-
2k-PEG	4.71 ± 0.01	244.5 ± 7.5	2k-PEG	1.56 ± 0.01	56.0 ± 0.5
5k-PEG	5.08 ± 0.01	5.4 ± 0.2	5k-PEG	2.26 ± 0.01	23.4 ± 0.2
20k-PEG	3.13 ± 0.01	0.2 ± 0.1	20k-PEG	1.76 ± 0.01	0.7 ± 0.1

### 2.2. Varying the Surface Coating of SPIONs Changes Its Toxicity Profile

**Figure 1:**
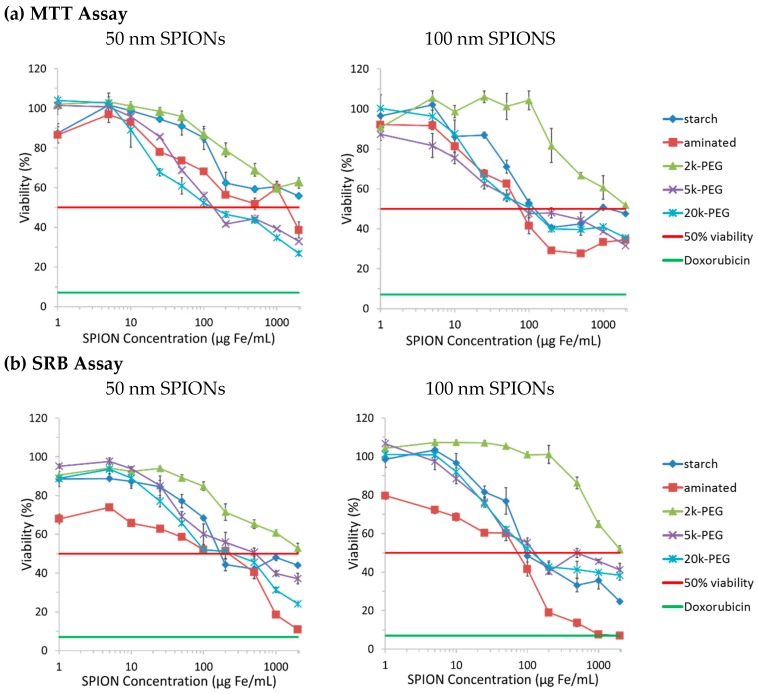
Cytotoxicity of superparamagnetic iron-oxide nanoparticles (SPIONs) in Chinese Hamster Ovary (CHO-K1) cells evaluated with the (**a**) tetrazolium dye 3-(4,5-dimethylthiazol-2-yl)-2,5-diphenyltetrazolium bromide (MTT) and (**b**) sulforhodamine B (SRB) assays. CHO-K1 cells were incubated with increasing concentrations of SPIONs for 72 h. The 50% viability of control cells (untreated) is represented by the red line. The viability of cells treated with 10 µM doxorubicin for 72 h (positive control) is shown by the green line.

Toxicity studies were conducted in CHO-K1 cells with increasing concentrations of SPIONs, using the MTT and SRB assays. Preliminary results showed a time-dependent cytotoxicity with the greatest difference among tested SPIONs observed at 72 h, which was fixed as the incubation time for further studies. Cytotoxicity profiles of tested SPIONs are reported in Figure 1, with cell viability expressed relative to untreated, control cells whose viability is set as 100%. Doxorubicin (10 µM) was employed as a positive control for these experiments (green line). The half maximal inhibitory concentration (50% viability or IC_50_) is indicated with a red line in all graphs. Mathematical regression was employed to approximate the IC_50_ values for each particle with both the MTT and SRB assays; IC50 values are reported in Table 3.

The MTT and SRB results showed similar cytotoxic tendencies. First, both 50 and 100 nm 2k-PEGylated SPIONs seemed to be better tolerated by CHO-K1 cells than the other coatings tested. The aminated 100 nm SPIONs, on the other hand, appeared to be the most toxic, except for the 50 nm SPIONs, as determined by their IC_50_ values. It should also be pointed out that the aminated 50 nm SPION sample is the one that had the lower than expected ζ potential, although the amine content was high. The connection, if any, between this and the observed lower toxicity is not clear. An IC_50_ value was not reached for 50 nm SPIONs with the starch and 2k Da PEG coatings, within the concentration range studied. In all cases, the 5k-PEG and 20k-PEG coated SPIONs were found to be significantly more toxic than particles coated with the 2k Da PEG. A comparison of IC_50_ values, for both the MTT and SRB assays, also indicates that the 100 nm SPIONs present greater cytotoxicity than the 50 nm SPIONs. A greater range of mean SPION sizes needs to be tested to determine whether this is a linear trend or if there are SPION sizes that would yield a minimal and/or maximal toxicity. 

Nanoparticle surfaces have been engineered to increase the *in vivo* circulation time [19,20,26]. Our results seem to indicate that PEG molecular weight and density could also be utilized to modulate the cytotoxicity of SPIONs. It should also be noted that surface chemistry plays a role in determining the composition of the protein corona that adsorbs onto the nanoparticle surface and, thus, also influences the uptake pathways followed by the particles [27].

**Table 3:** Half maximal inhibitory concentrations (IC_50_) of SPIONs incubated wtih CHO-K1 cells.

Samples	IC_50_ (µg Fe/mL)			
	50 nm SPIONs		100 nm SPIONs	
	MTT	SRB	MTT	SRB
Starch	Not reached	621	436	124
Aminated	1119	252	69	35
2k-PEG	Not reached	1867	1796	1563
5k-PEG	217	483	66	351
20k-PEG	136	217	147	205

### 2.3. Exposure Time Affects the Observed SPION Toxicity

**Figure 2:**
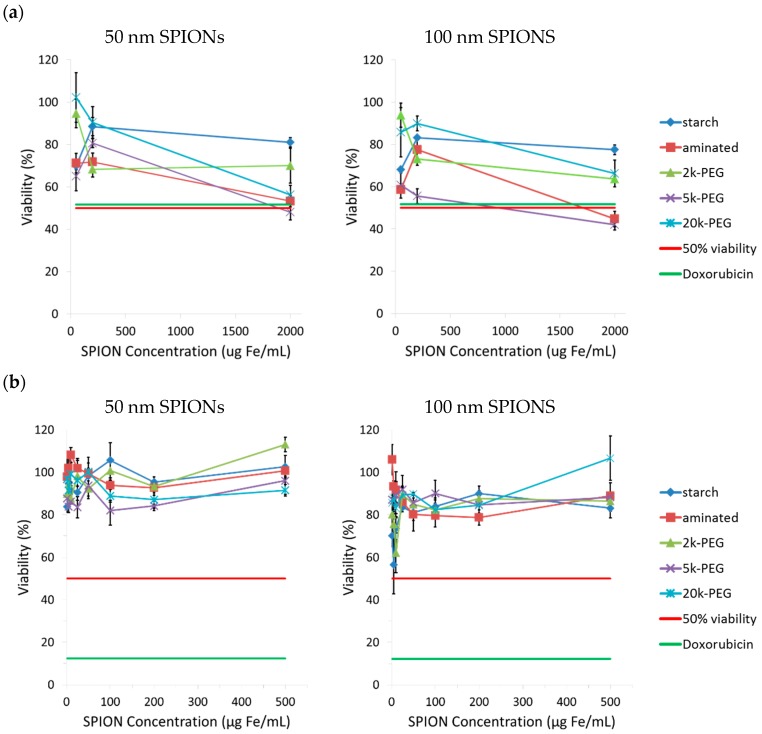
Assessment of the toxicity of SPIONs after, (**a**) 24 h incubation only, and (**b**) after 24 h incubation followed by 48 h of recovery in supplemented culture media; viability compared to untreated cells (control, 100% viability). The 50% viability of control cells (untreated) is represented by the red line. The viability of cells treated with 10 µM doxorubicin (positive control) is shown by the green line.

The *in vivo* blood half-life of SPIONs varies, depending on particle size and surface properties [20], from 2 h for dextran-coated 120–180 nm ferumoxide particles (Endorem^®^, Feridex^®^) to 24–36 h for dextran-coated 15–30 nm ferumoxtran particles (Sinerem^®^, Combidex^®^). In a recent study, the half-life of starch-coated SPIONs and those with 5k- and 2k-PEG coatings was found to be 0.12, 7.3, and 11.8 h, respectively, in male Fisher rats [20]. To mimic the potential *in vivo* contact time, CHO-K1 cells were incubated with SPIONs for a period of 24 h. The cytotoxicity was evaluated with the MTT assay after this contact time (Figure 2a). Alternatively, cells were also incubated with SPIONs for 24 h and then washed and cultured for another 48 h with fresh culture medium prior to analysis of cell viability (Figure 2b).

The viability of CHO-K1 cells varied between 100% and 50% when incubated 24 h with SPION concentrations up to 2000 µg Fe/mL (Figure 2a). However, when cells were allowed a 48 h recovery period in complete fresh culture medium, cell viability remained above 80% for concentrations up to 500 µg Fe/mL. A few studies have shown that internalized SPIONs can be divided in daughter cells during cell division, and consequently decrease the iron content per cell [22]. The presence of exocytosis has also been reported [28]. The attenuation of toxicity due to reduction of intracellular SPION concentration by these mechanisms is a possible explanation for the different tendencies seen in Figure 2a,b, but more focused studies are needed to elucidate the true effects of these processes. Conversely, the viability of cells treated with 10 µM doxorubicin dropped from 50% to 11% despite washings and incubation in fresh culture media, probably due to permanent cellular damage. The extrapolation of these results to *in vivo* conditions is difficult because CHO-K1 cells are an immortalized cell line, which is not the case for normal cells from ovaries. Our *in vitro* studies with CHO-K1 cells, however, has shown that the tested SPIONs cause reversible, dose- and time-dependent toxicity in rapidly dividing cells.

### 2.4. The Cellular Uptake Kinetics of SPIONs in Chinese Hamster Ovary (CHO-K1) Cells Is Dependent on Their Surface Coating

Despite the large number of cellular uptake studies that have been performed with SPIONs of various size and coating, it remains unclear which physicochemical characteristics provide optimal uptake in non-phagocytic cells. A saturable time- and concentration-dependent uptake has been demonstrated in astrocytes for dimercaptosuccinate-coated SPIONs [29], and charge-dependent uptake in non-phagocytic T-cells [30] for different sizes of dextran-coated SPIONs. Moreover, aminated aminosilane-coated SPIONs showed the highest iron uptake in six different cell lines, compared to silica, dextran, or bare SPIONs [12]. However, only few studies have studied the cellular uptake kinetics of SPIONs [12], and none for cells derived from a reproductive organ.

**Figure 3:**
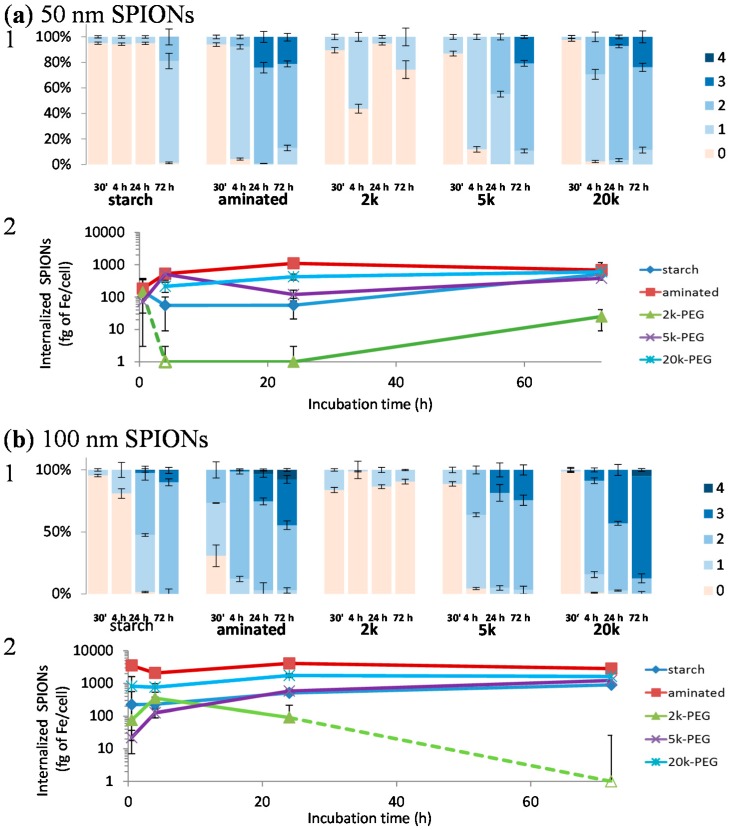
Kinetics of (**a**) 50 and (**b**) 100 nm SPION uptake by CHO-K1 cells, represented by (**1**) Average grading of particles, after 30 min, 4, 24, and 72 h of incubation. 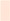 Grade 0 = no blue color, 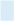 grade 1 = faint blue staining in cytoplasm, 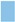 grade 2 = dense blue color in minor portions of cytoplasm (less than 50%), 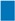 grade 3 = deep blue staining in most cytoplasm (>50%) and 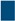 grade 4 = cell filled with intense, dark blue througout; and (**2**) Quantitfication of internalized iron using a ferrozine assay is reported below the histograms, with evolution of the quantitative uptake over time. Hollow markers indicate concentrations below the detection limit of the assay (*i.e.*, zeros).

Here, cell associated SPIONs were stained with Prussian blue and the intensity categorized into 5 grades, with grade 0 (orange/red, referring to the safranin staining of the cell membranes) showing no overt visible iron uptake in cells and grade 4 (dark blue, referring to the blue color obtained from Prussian blue staining of iron) showing maximum uptake. Average grades were assigned by grading all cells observed in 10 optical images taken at high magnification (400×). Thus, histograms in Figure 3 represent the uptake distribution and kinetics of SPIONs in CHO-K1 cells.

The pattern observed with staining of SPIONs in cells indicates that particles are found almost exclusively in the cytoplasm of cells (*i.e.*, not detectable in the nucleus). However, the staining employed does not differentiate between intracytoplasmic organelles, such as lysosomes. Once internalized, degradation of SPIONs through the lysosomal pathway is indeed considered the common metabolic pathway. The low pH environment in lysosomes is favorable for the solubilization of iron contained in SPIONs [31], releasing free iron ions in the cytoplasm.

While the uptake kinetics varied between tested SPIONs, the results indicate that surface properties have a greater influence on uptake than the particle size (at least between 50 and 100 nm SPIONs). Particles coated with 2k-PEG displayed the lowest rate of cellular uptake, followed by the starch SPIONs. Surprisingly, aminated, 5k- and 20k-PEG SPIONs showed similar uptake kinetics, although aminated and 20k-PEG SPIONs were taken up to a greater extent. It is interesting to note that the aminated SPIONs and the 5k- and 20k-PEGylated SPIONs all showed a similar trend in particle uptake, regardless of particle size. Each of these particles showed labeling of almost all cells within a 4 h incubation period and continued accumulation over 72 h. The 50 nm starch-coated particles, on the other hand, were only taken up by a few cells over 24 h and then all cells were lightly labeled after 72 h. The 100 nm starch-coated particles showed slightly faster kinetics with all cells labeled within the first 24 h and further accumulation over 72 h. SPIONs modified with the 2k Da PEG, on the other hand, exhibited very different cellular uptake kinetics with the CHO-K1 cells. While between 10%–15% of cells were labeled by 2k-PEG SPIONs in the first 30 min, this level of labeling remained fairly constant over the entire 72 h incubation period after which only 26% and 9% of cells were labeled for the 50 and 100 nm SPIONs, respectively. It should be noted that these results correlate well with the toxicity results presented in Figure 1. The kinetic studies were carried out with SPION concentrations of 10 µg Fe/mL, a concentration at which no appreciable toxicity is observed with the 2k-PEG SPIONs but at which toxicity begins to appear with the other particles. Ongoing studies are being carried out to clarify the processes governing the behavior of these particles and to determine the cause for this dramatic difference in their interaction with the cells.

In the literature, the presence of positively charged amine groups on the coating of polyvinyl alcohol (PVA)-functionalized SPIONs has been shown to increase uptake in human melanoma cells, compared to PVA alone, carboxylate-, or thiol-PVA groups [29]. Moreover, the uptake of SPIONs can also be affected by variations in the protein corona that coats the particle upon addition to culture media or exposure to serum proteins [27]. It has also been shown that SPION uptake can be inhibited by plasma proteins that coat the particles [30]. While the SPIONs showed varying rates of initial cellular uptake, over the 72 h period they appeared to approach the same maximum uptake regardless of surface coating; although the maximum approached was different for the 50 and 100 nm particles and the 2k-PEG SPIONs remained significantly lower than all other particles.

**Figure 4:**
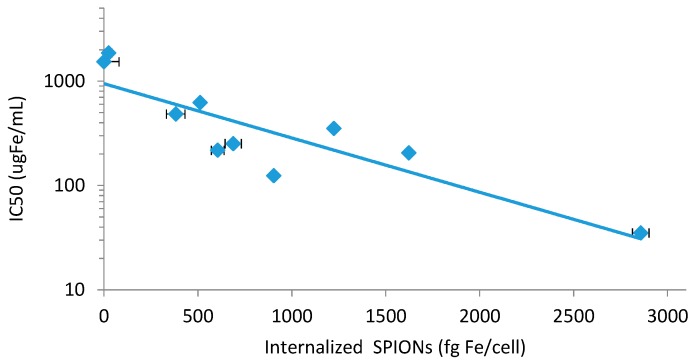
Correlation of the uptake kinetic of all type of SPIONs and their respective toxicity (represented by their IC50 values calculated from the SRB assay) after 72 h incubation with CHO-K1 cells.

To further determine whether the observed variation in SPION toxicity is due to differences in particle properties or because of differences in cellular uptake, the results from the kinetic study were correlated with those from the cytotoxicity studies. IC50 values from the SRB assay were used here as the MTT assay did not yield IC50 values for two particles (*i.e.*, starch and 2k-PEG coated). As seen in Figure 4, there is an exponential decrease (*i.e.*, greater toxicity) in measured IC_50_ values with increasing concentrations of internalized SPIONs, indicating that their internalization is an important step in the triggering of cell toxicity. The trend indicated that the toxicity of SPIONs, after 72 h incubation, approached the 35 µg Fe/mL IC_50_ value of the 100 nm, aminated SPIONs.

### 2.5. The Coating Affects the Generation of Reactive Oxygen Species (ROS) by SPIONs in CHO-K1 Cells

**Figure 5:**
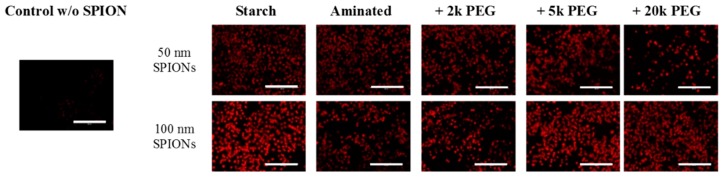
ROS production (red) in CHO-K1 cells following incubation with SPIONs was confirmed with the H2DCFDA assay. Control cells underwent the same assay but were not exposed to SPIONs. Scale bar = 200 µm.

The production of reactive oxygen species (ROS) is often reported as the source of nanoparticle-associated toxicities [31,32]. Consequently, to further evaluate the mechanism of SPION cytotoxicity, H2DCFDA assay was performed to detect the generation of intracellular ROS. While it was clear that SPIONs did indeed generate significant ROS within CHO-K1 cells, in comparison to control, the study was unable to detect differences between the SPION particles due to signal saturation (Figure 5). Additional studies must be conducted to better quantify this process and its contribution to the observed cytotoxicity.

### 2.6. Surface Properties Are Key to Determining the Cytotoxicity of SPIONs in CHO-K1 Cells

In this study, the uptake kinetics and cytotoxicity of starch, aminated and PEGylated SPIONs were determined in CHO-K1 cells. Taken together, the results suggest that the cytotoxicity of the tested SPIONs were due to the generation of ROS upon internalization of the particles. Since surface properties were found to significantly affect the kinetics and extent of internalization, the toxicity of SPIONs were found to be most dependent on the surface modification. It should, however, also be noted that toxicity of 100 nm SPIONs were generally greater than that of the corresponding 50 nm SPION with the same coating. This is interesting as at a given SPION concentration, on a mass of iron basis, the total surface area of the 50 nm particles will be expected to be approximately four-fold greater than that of the same iron mass of 100 nm SPIONs; although the specific surface area of the 100 nm SPIONs is greater on a per particle basis. Therefore, although surface effects seem to drive SPION toxicity, the effects of particle size cannot be ignored.

## 3. Experimental Section

### 3.1. Surface Modification of Starch-Coated SPIONs

Two different sizes of starch magnetite core nanoparticles (FluidMAG-D, autoclaved aqueous dispersion) were purchased from chemicell GmbH (Berlin, Germany). Manufacturer specifications indicated average hydrodynamic diameters of 50 and 100 nm, respectively, for the two SPIONs. Cross-linking of the starch coating, amination, and PEGylation of the particles was performed according to methods described by Cole *et al.* [20]. The physical properties of the SPIONs were confirmed to be in accordance with results published by Cole *et al.*, to which the readers are referred to for more details, but are not reproduced here for brevity. Briefly, starch moieties on the surface of iron-oxide nanoparticles were crosslinked with epichlorohydrin at room temperature for 24 h. The dialyzed product was then aminated with ammonium hydroxide (28%–30%) for 24 h at room temperature. After a second dialysis against water, one quarter of the product was labeled as aminated SPIONs. The remaining product was PEGylated using m-PEG-NHS (Nanocs, New York, NY, USA) in pH 8 phosphate buffer/DMSO solution incubated overnight. mPEG molecules with molecular weights of 2k, 5k, and 20k Da were used in this study. Ultrafiltration of the product was performed using a 10,000 molecular weight cut-off (MWCO) Slide-A-Lyzer dialysis cassette until PEG was no longer detectable in the washes using the Barium-Iodine assay described below.

### 3.2. Characterization of Starch-Coated, Aminated and PEGylated SPIONs

Hydrodynamic diameter and ζ potential of each SPION sample was determined by Dynamic Light Scattering (Zeta Sizer Nano-ZS from Malvern Instruments, Malvern, UK). SPIONs were dispersed in DI water or in phenol red-free culture media (Ham’s F-12K Ultra Pure, Crystalgen, NY, USA), supplemented with fetal bovine serum (10%) and antiotics (penicillin/streptomycin 1%). All DLS measurements were performed at 37 °C.

Measurement of amine content was performed using the fluorescamine assay [20]. A standard curve was established using solutions with known ethanolamine concentrations, starch SPIONs, and fluorescamine.

A barium iodine assay was used to quantify the average concentration of PEG attached on SPIONs. SPIONs were first dissolved in 1.4 HCl overnight prior to analysis. Standard curves were generated with starch-coated SPIONs (50 or 100 nm) and varying concentrations of free PEG molecules (2k, 5k, or 20k Da PEG).

All measurements for the physicochemical characterization of SPIONs were performed in triplicate, and results are expressed as the mean and standard error of mean. 

### 3.3. Cell Line

The CHO-K1 cell line was obtained from Rajesh Amin, Department of Drug Discovery and Development, Auburn University, Auburn, AL, USA. Authentication of the cell line was complimentary confirmed by the National Institute of Standards and Technology, using a multiplex PCR assay (non commercial assay). Cells were maintained in Ham’s F-12K Nutrient Mixture with l-gluthamine (Corning cellgro, Manassas, VA, USA), supplemented with 10% fetal bovine serum and 1% penicillin/streptomycin, incubated at 37 °C in 5% CO_2_.

### 3.4. Cell-Viability Assays

Cells were plated into 96-well flat-bottom plates at a starting density of 10^4^ cells per well. After an equilibration time of 12 to 24 h, until they reached 70%–80% confluency, culture media was removed and cells were treated in triplicate with increasing concentrations (1, 5, 10, 25, 50, 100, 200, 500, 1000, and 2000 µg Fe/mL) of the 10 different SPIONs suspended in culture media. Cell viability was then determined with 3-(4,5-dimethylthiazol-2-yl)-2,5-diphenyltetrazoliumbromide (MTT assay; AMRESCO, Solon, OH, USA) and sulforhodamine B (SRB; CytoScan^TM^ SRB Cytotoxicity Assay kit, G Biosciences, St. Louis, MO, USA) assays. The MTT assay determines the viability of cells according to their metabolic activity, whereas the SRB assay non specifically detects the total protein content in cells. 

Briefly, the MTT assay was conducted by incubating cells with a 2 mg/mL solution of MTT (50 µL) for 4 h. The supernatant was then aspirated and 150 µL of dimethyl sulfoxide (DMSO) was applied to the cells to solubilize the formed formazan crystals. Plates were agitated and then centrifuged (3000 rpm for 10 min) to pellet the SPIONs. Finally, 100 µL of the supernatant was collected and transfered into a clean 96-well flat-bottom plate. Absorbance was measured at 540 nm using a microplate reader (SpectraMax i3 multi-mode platform, Molecular Devices, Sunnyvale, CA, USA).

The SRB assay was conducted in accordance to instructions provided with the commericial kit, except that cells were washed twice with 1× PBS prior to fixing of cell. Absorbance was measured at 540 nm using a microplate reader (SpectraMax i3 multi-mode platform, Molecular Devices).

#### 3.4.1. Overall Toxicity after 72 h of Incubation

The overall toxicity of SPIONs were tested using the cell viability assay. CHO-K1 cells were incubated 72 h with particles and viability was assessed with MTT and SRB assays. For both assays, a positive control was also added to verify the response of the cell line (Doxorubicin 10 µM) [33]. Results are reported as the percentage of viable cells compared to untreated cells (negative control, by definition considered 100% viable). Cell-viability results are presented as means ± standard error of the mean (SEM) of triplicates for each concentration tested. Results are reported in Figure 1.

#### 3.4.2. Toxicity in Conditions that Mimic *in Vivo* Contact Time of SPIONs

To mimic potential contact time of SPIONs in the body, CHO-K1 cells were seeded in 96-well plates at 10^4^ cells/well and equilibrated overnight. They were incubated for 24 h at 37 °C with SPIONs at the following concentrations: 50, 200, and 2000 µg Fe/mL. Alternatively, CHO-K1 cells were incubated with 1, 5, 10, 25, 50, 100, 200, and 500 µg Fe/mL, then washed and cultured in fresh culture medium for another 48 h. Cytotoxicity was evaluated with the MTT assay (described above). Doxorubicin (10 µM) was employed as a positive control for the experiment (wells containing Doxorubicin were also washed after 24 h). Results are presented in Figure 2.

### 3.5. Characterization of SPION Uptake by CHO-K1 Cells

#### 3.5.1. Uptake Kinetics

To determine the uptake characteristics of tested SPIONs in CHO-K1 cells, qualitative and quantitative measurements were performed based on the detection of iron in SPIONs. First, the presence of iron inside cells was visually detected by staining iron with Prussian blue. The presence of iron in cells was also quantified using a ferrozine assay, as previously described [34].

#### 3.5.2. Direct Microscopic Examination

Briefly, CHO-K1 cells were plated in a 24-well plate at a starting density of 5 × 10^4^ cells per well. After overnight equilibration, the culture media was removed and the cells incubated with SPIONs at 10 µg Fe/mL, for 30 min, 4, 24, or 72 h. Cells were then washed twice with 1× PBS, and stained with a Prussian blue staining solution (2% potassium ferrocyanide and 2% hydrochloric acid, Electron Microscopy Sciences, Hatfield, PA, USA) for 20 min at room temperature. After washing five times with PBS 1×, cells were then counterstained with safranin for 2 min. The cell membranes were stained red/orange, whereas iron was stained in blue. To ensure good representation of samples, uptake was assessed with 10 images acquired at 400× magnification from various areas of two different wells.

A scoring system (detailed in Table 4) was established to characterize the amount of iron internalized in each cell. This scoring system is derived from a protocol described in Human and Veterinary Medicine to assess hemosiderosis in alveolar macrophages [35,36]. All entire cells found within an acquired image were graded. Sample names were blinded prior to analysis but 10 pictures from one sample type were graded at the same time. Results are presented in Figure 3.

**Table 4:** Scheme for scoring of cellular uptake of SPIONs. The following set of images are illustrative of the grading used to quantify SPION uptake by CHO-K1 cells. Cells were stained with Prussian blue (blue spots that indicate presense of iron) and Safranin (pinkish coloration of cellular membranes). Cells shown are representative of those seen at 400× magnification. A scale bar is not provided since these are only representative cell images that were digitally extracted from original images that contained many cells with varying SPION uptake.

Grade 0	Grade 1	Grade 2	Grade 3	Grade 4
No blue color	Faint blue staining in cytoplasm	Dense blue color in <50% of cytoplasm	Deep blue staining in >50% of cytoplasm	Cell cytoplasm filled with intense dark blue
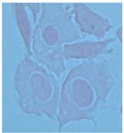	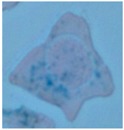	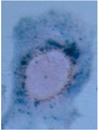	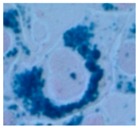	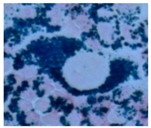

##### Determination of Internalized Iron Concentration Using the Ferrozine Assay

The amount of internalized iron was quantified with a ferrozine-based assay [34], normalized against the number of cells and the iron physiologically present in cells under normal conditions. A preliminary study was performed to establish a standard curve that correlates the CHO-K1 cell number and the result of the Bradford assay (colorimetric assay). Results of iron taken up by cells were also in accordance to previous evaluations (data shown in Figure 3).

### 3.6. Overall Reactive Oxygen Species (ROS) Generation

The CM-H2DCFDA kit purchased from Invitrogen was used for the measurement of overall ROS levels. Briefly, 2.5 × 10^4^ cells were plated in 24-well plates in duplicate. After overnight equilibration, cells were treated with tested SPIONs for 48 h at a final concentration of 200 µg Fe/mL. Prior to the end of treatment, culture media was remove and DCFDA in PBS 1× was added to each well at a final concentration of 7 µM and incubated in the dark at 37 °C for 45 min. After treatment, the fluorescence intensity of CM-H2DCFDA in cells was evaluated using a fluorescence cell imaging system (Evos^®^, ThermoFisher Scientific, Waltham, MA, USA). Data shown in Figure 5.

## 4. Conclusions

CHO-K1 cells internalize SPIONs with various types of coating in a concentration-dependent manner. The toxicity profiles observed for the SPIONs suggest the plausibility of saturable uptake kinetics, as previously described in astrocytes [37]. Several physicochemical parameters are described in literature as potential variables affecting uptake and, consequently, cytotoxicity. Assessing and understanding their impact is vital to the development of novel, targeted cellular delivery carriers. While size is an important parameter, the effects were less than expected, although only two mean particle sizes with potential overlapping size distributions were studied. Surface charge can have a significant impact on the rate and route of SPION uptake. It is generally accepted that the negative charge of cell plasma membranes leads to strong interactions with positively charged nanoparticles and their increased uptake [38]. In this study, positively-charged, aminated SPIONs were indeed found to be taken up the most rapidly and to the greatest extent, followed by 20k-PEGylated SPIONs and 5k-PEGylated SPIONs. Surprisingly, 2k-PEGylated SPIONs had a positive surface charge similar to that of the 20k- and 5k-PEGylated SPIONs but were taken up to a lesser extent. Cytotoxicity results showed that the 2k-PEG SPIONs were the best tolerated at all concentrations studied. Similarly, starch SPIONs seemed to be better tolerated at concentrations below 100 µg Fe/mL compared to the 5k- and 20k-PEGylated SPIONs, possibly attributed to lower uptake induced by their slightly negative charge. The 2k-PEG coating, which yielded the densest surface coating of SPIONs among the PEGs tested, showed the least degree of cytotoxicity against CHO-K1 cells. Overall, the correlation between surface properties of SPIONs and their cellular uptake, and the correlation between their uptake and cytotoxicity, suggests a means to modulate the disposition and biocompatibility of nanoparticles. Further studies need to be conducted to better characterize the nature of the interaction between these particles and cells, and also to elucidate their subcellular localization. It will also be important to study their effects on the reproductive system using *in vivo* models.
